# Methanogen Productivity and Microbial Community Composition Varies With Iron Oxide Mineralogy

**DOI:** 10.3389/fmicb.2021.705501

**Published:** 2022-02-18

**Authors:** Hayley J. Gadol, Joseph Elsherbini, Benjamin D. Kocar

**Affiliations:** ^1^Department of Civil and Environmental Engineering, Massachusetts Institute of Technology, Cambridge, MA, United States; ^2^Graduate Program in Microbiology, Massachusetts Institute of Technology, Cambridge, MA, United States; ^3^Environmental Laboratory, U.S. Army Engineer Research and Development Center, Vicksburg, MS, United States

**Keywords:** methanogenesis, iron reduction, *Geobacter*, *Methanosarcina*, iron (oxyhydr)oxides

## Abstract

Quantifying the flux of methane from terrestrial environments remains challenging, owing to considerable spatial and temporal variability in emissions. Amongst a myriad of factors, variation in the composition of electron acceptors, including metal (oxyhydr)oxides, may impart controls on methane emission. The purpose of this research is to understand how iron (oxyhydr)oxide minerals with varied physicochemical properties influence microbial methane production and subsequent microbial community development. Incubation experiments, using lake sediment as an inoculum and acetate as a carbon source, were used to understand the influence of one poorly crystalline iron oxide mineral, ferrihydrite, and two well-crystalline minerals, hematite and goethite, on methane production. Iron speciation, headspace methane, and 16S-rRNA sequencing microbial community data were measured over time. Substantial iron reduction only occurred in the presence of ferrihydrite while hematite and goethite had little effect on methane production throughout the incubations. In ferrihydrite experiments the time taken to reach the maximum methane production rate was slower than under other conditions, but methane production, eventually occurred in the presence of ferrihydrite. We suggest that this is due to ferrihydrite transformation into more stable minerals like magnetite and goethite or surface passivation by Fe(II). While all experimental conditions enriched for *Methanosarcina*, only the presence of ferrihydrite enriched for iron reducing bacteria *Geobacter*. Additionally, the presence of ferrihydrite continued to influence microbial community development after the onset of methanogenesis, with the dissimilarity between communities growing in ferrihydrite compared to no-Fe-added controls increasing over time. This work improves our understanding of how the presence of different iron oxides influences microbial community composition and methane production in soils and sediments.

## Introduction

Methane is an important greenhouse gas, possessing a global warming potential about 28 times greater than carbon dioxide over a 100-year period (Jain et al., 2000; IPCC, 2013). In natural systems, it is often unclear why methane emissions vary across or between landscapes and over time. Many previous studies, especially in soils, have focused mainly on physical factors like temperature and humidity on methane emissions (Oertel et al., 2016); the impact of varying mineralogical and chemical properties of these environment is less clear. A thorough understanding of how microbial processes are affected by soil/sediment chemistry and mineralogy, is necessary for understanding net methane production in natural environments.

Iron(III) (oxyhydr)oxide minerals, hereafter referred to as iron oxides, are widely found in environmental systems (Cornell and Schwertmann, 2003). These minerals are strong sorbents of nutrients (Sibanda and Young, 1986; Strauss et al., 1997) and potentially toxic contaminants (Bowell, 1994; Brown and Parks, 2001), and they also serve as terminal electron acceptors for microorganisms under anaerobic conditions (Kappler and Straub, 2005; Weber et al., 2006; Lentini et al., 2012). There are a range of iron oxide minerals with differing crystallinities, surface areas, and thermodynamic stabilities in soils and sediments (Cornell and Schwertmann, 2003). Iron-reducing bacteria are able to reduce various iron oxide minerals, including ferrihydrite (nominally Fe(OH)_3_) (Lovley and Phillips, 1988), goethite (α-FeOOH) (Liu et al., 2001), and hematite (Fe_2_O_3_) (Yan et al., 2008). Additionally, magnetite (Fe_3_O_4_), a mixed Fe(II)/Fe(III) mineral, can be produced as a byproduct of ferrihydrite reduction (Lovley et al., 1987), and be further reduced to Fe^2+^_(aq)_ (Kostka and Nealson, 1995). Due to their differing physicochemical properties, some of these poorly crystalline minerals, such ferrihydrite or amorphous iron oxides, are more susceptible to microbial iron reduction than those that are more highly crystalline, including goethite and hematite (Lovley and Phillips, 1986; Phillips et al., 1993; Roden and Zachara, 1996; Bray et al., 2017). This can also lead to pronounced differences in the microbial communities that associate with different iron oxide minerals (Lentini et al., 2012).

Competition between microbes possessing different metabolic capabilities, like iron reduction or methanogenesis, has been summarized in terms of a “thermodynamic ladder” of terminal electron acceptors (Champ et al., 1979; Froelich et al., 1979; Bethke et al., 2011). When microbes compete for the same electron donor, such as acetate, those that use the most energy-yielding electron acceptors should outcompete those that use lower energy-yielding terminal electron acceptors. This leads to redox zonation of different terminal electron acceptors in the environment, where oxygen is consumed first, followed by nitrate, manganese, iron, and sulfate; the methanogenesis zone forms where all more favorable energy-yielding reactions have been exhausted, typically at the bottom of a profile (Canfield and Thamdrup, 2009). Hence, in the presence of poorly crystalline iron(III) oxides, such as ferrihydrite, acetotrophic iron reduction should out-compete acetoclastic methanogenesis (Bethke et al., 2011). However, reduction of more crystalline iron oxides, like hematite and goethite, may not outcompete methanogenesis due to their greater thermodynamic stabilities and therefore lower susceptibilities to microbial iron reduction (Bethke et al., 2011). Thus, despite high concentrations of oxidized Fe, methanogens may outcompete iron-reducing bacteria in the presence of well-crystalline iron oxides.

Indeed, many studies have shown that iron reduction outcompetes methanogenesis in wetlands and soils when iron is present as a poorly crystalline iron oxide (Sahrawat, 2004; Laanbroek, 2010). However, there are also many instances where methanogenesis and iron reduction occur concomitantly (Slomp et al., 2013; Vigderovich et al., 2019), or where methanogenesis dominates in zones of ferruginous lakes with persistent iron oxides (Lambrecht et al., 2020; Friese et al., 2021). A decreased relative ability of iron-reducers to out-compete methanogens in the presence of well-crystalline iron oxides may explain overlapping zones of iron reduction and methanogenesis in the environment.

The influence of iron oxide crystallinity on competition between iron reduction and methanogenesis has already been studied under a number of conditions. Work by Aromokeye et al. (2018) with marine sediment incubations showed that iron reducers did not outcompete methanogens at 10°C in the presence of hematite and magnetite. This agrees well with thermodynamic calculations, which predict ferrihydrite reduction should outcompete methanogenesis while reduction of more crystalline iron oxides would not. However, iron reduction did outcompete methanogenesis at 4°C and the crystalline iron oxides substantially stimulated methanogenesis relative to controls at 30°C (Aromokeye et al., 2018). Additionally, acetate-limited wetland sediment incubations of amorphous and crystalline iron oxides led to similar outcompeting of methanogens by iron reducers when the iron oxides were added at similar loadings by surface area (Roden and Wetzel, 2003). This demonstrates that thermodynamic predictions alone are not enough to explain interactions between iron-reducing bacteria and methanogens.

Furthermore, in some environments carbon may not be the limiting factor for iron reduction, such as in the soils of the Artic Coastal Plain (Miller et al., 2015) and a landfill-leachate polluted aquifer in Denmark (Albrechtsen et al., 1995). An excess of carbon may allow methanogenesis to persist concurrently with iron reduction. Work by Teh et al. (2008) in tropical forest soils showed that the addition of acetate increased methanogenesis by 67 times while only doubling iron reduction. Dubinsky et al. found that addition of carbon to soil slurries increased the amount of methane production relative to iron reduction, despite an increase in the total rates of both processes (Dubinsky et al., 2010). This suggests that an excess of acetate relative to iron also plays a role in governing competition between iron reducers and methanogens regardless of iron oxide crystallinity.

Accordingly, the goal of this study is to understand how different iron oxide minerals affect methane production by influencing competition between iron reducing bacteria and methanogens under conditions not limited by electron donor (acetate). Both chemical and microbial community analysis are used to understand the balance between methane production and iron reduction in the presence of poorly crystalline ferrihydrite compared to well-crystalline hematite and goethite. Thermodynamic calculations under a range of acetate concentrations are used to predict conditions under which iron reducers should outcompete methanogens, and the resulting predictions are compared to the chemical and microbial community results obtained in incubation experiments. The results of this work improve our understanding of interactions between iron-reducing bacteria and methanogens, and consequently methane and iron cycling, in natural environments, including soils and sediments.

## Materials and Methods

### Thermodynamic Calculations

The standard state ΔG°_rxn_ for each considered metabolic reaction was tabulated based on published ΔG°_f_ values for all reactants and products. These values are summarized in Supplementary Table 1. The ΔG°_rxn_, at 298K and 1 M activities of all reactants and products, is calculated based on Equation 1.


(1) △G°rxn=△G°products-△G°reactants

These ΔG°_rxn_ values were then converted to ΔG_rxn_ values at specific environmental conditions using Equation 2.


(2) △Grxn=△G°rxn+RTlnQ

where, *R* = 8.314 J*mol^–1*^K^–1^, T = 298K and Q is the reaction quotient calculated based on the reaction in Equation 3 (where A and B are reactants and C and D are products) using Equation 4.


(3) aA+bB→cC+dD

(4) $$Q=\frac{{\left[C\right]}^{c}{\left[D\right]}^{d}}{{\left[A\right]}^{a}{\left[B\right]}^{b}}$$

This was done for a number of acetate consuming reactions, including iron reduction and methanogenesis, as listed in Table 1.

**TABLE 1:** ΔG°_rxn_ values for relevant iron-reducing and methanogenesis reactions under standard state conditions.

Metabolism	Reaction	ΔG°_rxn_ (kJ/mol)	ΔG_rxn_ (kJ/mol) (100 uM acetate)	ΔG_rxn_ (kJ/mol) (10 mM acetate)
Magnetite reduction	4 Fe_3_O_4_ + CH_3_COO^–^ + 23 H^+^ → 12 Fe^2+^ + 2 HCO_3_^–^ + 12 H_2_O	−546.47	166.63	155.22
Ferrihydrite reduction	8 Fe(OH)_3_ + CH_3_COO^–^ +15 H^+^ → 8 Fe^2+^_(aq)_ + 2 HCO_3_^–^ + 20 H_2_O	−510.83	–48.74	–60.15
Goethite reduction	8 FeOOH + CH_3_COO^–^ +15 H^+^ → 8 Fe^2+^_(aq)_ + 2 HCO_3_^–^ + 12 H_2_O	−370.99	91.10	79.69
Hematite reduction	4Fe_2_O_3_ + CH_3_COO^–^ +15 H^+^ → 8 Fe^2+^_(aq)_ + 2 HCO_3_^–^ + 8 H_2_O	−347.87	114.22	102.81
Methanogenesis	CH_3_COO^–^ + H_2_O → CH_4(aq)_ + HCO_3_^–^	−14.68	–31.79	–43.20

*All values for acetate-consuming reactions are calculated per mol of CH_3_COO^–^. Reactions are listed from most to least favorable at standard conditions. ΔG_rxn_ values were calculated at pH 7 using (HCO_3_^–^) = 0.01, (Fe) = 0.001, and [CH4(aq)] = 0.00001.*

### Iron Oxide Preparation and Characterization

Iron oxide minerals were prepared using methods previously described (Cornell and Schwertmann, 2003). The authors have used these methods previously to successfully synthesize iron oxides and characterized the resulting products with consistent results between batches (Gadol et al., 2017; Chen and Kocar, 2018). Goethite was prepared by slowly adding 100 mL of a 1 M ferric nitrate solution to 180 mL of 5 M sodium hydroxide while stirring in 1 L polypropylene container. The sodium hydroxide solution was made from a freshly opened sodium hydroxide container to limit effects resulting from base carbonation, such as hematite contamination. This mixture was then diluted to about 1 L using deionized water and placed into a 70°C oven for 60 h. Hematite was prepared by slowly adding 300 mL of a 1 M sodium hydroxide solution to 500 mL of a 1 M ferric nitrate solution in a polypropylene container while stirring. Then, 200 mL of 1 M sodium bicarbonate was added to the mixture before placing it into an oven at 98°C for 5 days. 2-line Ferrihydrite was prepared by stirring 40 g of FeCl_3_6H_2_O into 3 L of deionized water. The pH of this solution was titrated periodically to pH 8 for about 2 h.

After syntheses were complete, each mineral was rinsed to remove sorbed electrolytes. This was achieved through centrifugation at approximately 3000 RPM for 30 min, followed by decantation of the supernatant and replacement with deionized (>18 MΩ) water, and vigorous shaking to resuspend the mineral pellet. This process was repeated three to five times. The color of hematite and goethite, and the HCl-extractable Fe concentrations from the first time point of the incubation experiments (as explained in Experimental Setup) is shown in Supplementary Figure 1.

### Field Site and Sampling

Experiments were inoculated with sediments obtained from Upper Mystic Lake, a eutrophic, dimictic, kettle hole lake in Arlington, MA, United States. This lake sediment was chosen because it has known methane ebullition in the sediment (Scandella et al., 2011; Varadharajan and Hemond, 2012; Delwiche and Hemond, 2017). Methane concentrations in the hypolimnion between 200 and 700 μM have been reported (Peterson, 2005), and a porewater methane concentration of 4 mM has been observed (Varadharajan, 2009). Sediment porewater DOC concentrations between 1.5 and 6 mM have been reported (Chin and Gschwend, 1991). Many other biogeochemical properties of the lake, including seasonal iron and nitrogen cycling, and gradients in microbial communities, are also well-studied (Senn and Hemond, 2004; Arora-Williams et al., 2018).

Sediments were collected from the southwest side of the lake on 26 July 2018, as shown in Supplementary Figure 2. The water depth at this sampling location is approximately 15 m, which is deeper than the thermocline depth of about 4–8 m on this date (Delwiche et al., 2020). Sediments were collected using an Ekman dredge and were transferred into a Mason jar filled to the top with sediment to ensure there was no headspace. The capped and sealed Mason jar with sediment was refrigerated until use.

### Experimental Setup

Incubations were prepared in triplicate in 500 mL serum bottles (Wheaton) sealed with butyl septa and aluminum crimp seals, and bottles were covered with aluminum foil to keep experiments dark. All incubations contained 1mM NH_4_Cl, 1mM KH_2_PO_4_, 0.5 mM MgCl_2_, 0.5 mM CaCl_2_, 5mM NaHCO_3_, 10mM HEPES, 0.05% Bacto yeast extract, 64 μg/L Na_2_WO_4_, and 1mL/L of a trace element solution (Fermentation Trace Metals Solution, Teknova). Experiments were also supplemented with 10 mM sodium acetate as an electron donor and carbon source. Hematite, goethite, or ferrihydrite was added to experiments with the exception of the control bottles in amounts equivalent to 10 mM as Fe(III). The pH of each experimental setup was adjusted to 7.00 (±0.05) using hydrochloric acid. Total solution volumes in all experiments were 200 mL.

**FIGURE 1:**
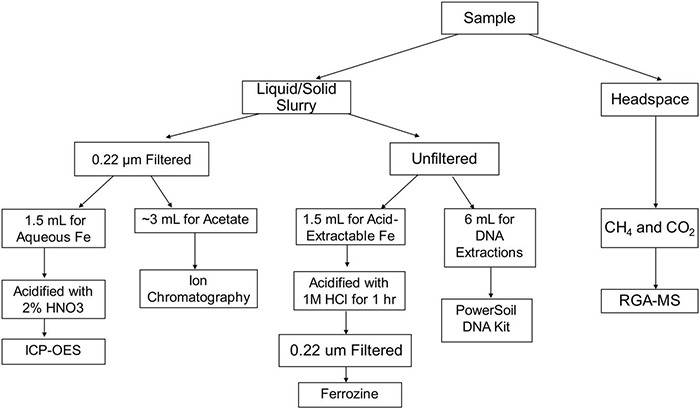
Visual representation of sampling protocol and analyses.

Before inoculation, all bottles were sparged with nitrogen gas for 30 min. Incubations were inoculated with approximately 0.5 mL of wet sediment to serve as a natural methanogenic microbial community from Upper Mystic Lake (UML). This was done in an anaerobic glove bag using a needle and syringe into the sealed serum bottles. The bottles were then incubated with shaking at 27°C for about 30 days. Time series data on methane and iron speciation was collected throughout the 30 days. DNA was collected for 16S rRNA community analysis after 9, 18, and 29 days. All sampling was conducted in an anaerobic glove bag with an atmosphere of N_2_ and H_2_ [98.5:∼1.5 (v/v)], and H_2_ was purposely kept at the minimum concentration recommended by the manufacturer to limit hydrogen introduction into the incubation experiments. A visual representation of the sampling process discussed in the following sections is shown in Figure 1.

### Methane Measurement

All gas measurements were made using a residual gas analyzer mass spectrometer/membrane inlet mass spectrometer (RGA-MS/MIMS) (Hiden Analytical) in the RGA configuration. Measurements were made using the Faraday detector and the MASsoft software was used in multiple ion detection (MID) mode. Sample tubing connected to the RGA instrument was extended into the glove bag through a port, so serum vial headspace sampling could be performed within the glove bag. An 18-gauge needle was attached to the end of the sample tubing and pierced through the serum bottle septum for each sample measurement.

To measure as much of the total gas in the sample as possible, *m/z* of 2 (H_2_), 15 (CH_4_), 16 (CH_4_/O_2_), 18 (H_2_O vapor), 28 (N_2_), 32 (O_2_), and 44 (CO_2_) were measured in each sample. Samples were measured for approximately 3 min, with the instrument producing values approximately every 15–20 s. After 3 min, the readings were typically stable for at least the last few measurements. Thus, the last five readouts were averaged to determine the m/z signal composition for each particular sample. All sensitivity factors were set to 1 and calibration was performed manually.

Calibrations were performed for CH_4_ and CO_2_ relative to N_2_. CH_4_, CO_2_, and N_2_ made up 96% or more of the total observed signal in all samples. Ultra-high purity methane and food-grade carbon dioxide mix (10% CO_2_/90%N_2_) were diluted with industrial-grade N_2_ to produce standards. For CH_4_, *m/z* of 15 was used for all calibrations while *m/z* of 44 was used for CO_2_. Resulting linear calibration curves were used in place of the sensitivity factors provided by the instrument manufacturer and used to correct the measured ratios of CH_4_:N_2_ and CO_2_:N_2_ to actual ratios in each sample. The fractions of CH_4_ and CO_2_ in the headspace were then determined using the following equations:


(5) $$F_{CH_{4}}=\frac{R_{CH_{4}}}{R_{CH_{4}}+R_{CO_{2}}+1}$$

(6) $$F_{CO_{2}}=\frac{R_{CH_{4}}}{R_{CH_{4}}+R_{CO_{2}}+1}$$

where, F_CH4_ is the fraction of CH_4_ in the headspace, F_CO2_ is the fraction of CO_2_ in the headspace, R_CH4_ is the measured calibration-corrected ratio of CH_4_:N_2,_ and R_CO2_ is the measured calibration-corrected ratio of CO_2_:N_2_ These fractions were then converted into moles methane produced at each time point as follows. Because it could not be determined directly, it was assumed that the total pressure in the headspace was 1 atm (which is likely an underestimate due to production of CO_2_ and CH_4_). Thus, the fraction of methane was assumed to be the methane partial pressure in atm. The partial pressure was then converted into moles using the ideal gas law at 298 K. The headspace was assumed to be the initial headspace at T_0_ (300 mL) plus the additional headspace created by withdrawing ∼6 mL of liquid sample at each time point. This value is the moles of methane present in the sample at the time of sampling.

Because RGA sampling withdrew a substantial amount of gas from the serum vial headspace, a correction was calculated to account for methane removed during previous samplings. A sample volume withdrawal rate could not be measured directly but was estimated using a 1 mL syringe. The amount of time it took to sample an entire 1 mL syringe was determined to be 26 s. Thus, for a total sampling time of 3 min, approximately 7 mL of sample was consumed. For each measurement, the total moles of methane present were added to the moles of methane in 7 mL of the headspace as determined in the measurement, plus any methane removed in previous time points calculated in the same way. At the first time point (T_0_), only the methane present at that time was used as total methane produced since no sample had been previously withdrawn.

### Liquid Sampling and Measurement

Samples for all aqueous analyses were collected in an anaerobic glove bag using a 5 mL syringe with a 22-gauge needle. During collection, syringes were filled completely to achieve a sample collection volume of ∼6 mL. 1.5 mL of this sample was placed into 1.5 mL of 2M hydrochloric acid (HCl) for HCl-extractable Fe analyses in centrifuge tubes. This sample was filtered after approximately 1 hour using 0.22 μm PES syringe filters into new centrifuge tubes. The remaining sample was filtered immediately at the time of sampling and 1.5 mL of this filtered sample was added to 1.5 mL of 4% nitric acid for aqueous Fe analysis. The remaining filtered sample was placed into a freezer to be preserved for acetate measurement.

HCl-extractable Fe(II) and total HCl-extractable Fe concentrations were measured using the Ferrozine method (Stookey, 1970) on a UV-Vis Spectrophotometer (Beckman DU800). Total HCl-extractable Fe concentrations were determined using hydroxylamine hydrochloride as a reducing agent. HCl-extractable Fe(II) concentrations were determined without the addition of hydroxylamine hydrochloride. Aqueous Fe concentrations were determined using inductively coupled plasma-optical emission spectroscopy (ICP-OES).

The remaining filtered liquid sample (∼1.5 mL) was used for acetate analysis by ion chromatography (IC, Dionex Integrion). Samples were analyzed using AS11-HC-4m analytical and guard columns for anion analysis with potassium hydroxide as the eluent. Peak area determination was performed using the Chromeleon software. Calibration curves based on peak areas in standards were fit according the form in VandenBoer et al. (2012) using the scipy.optimize.curve_fit (Virtanen et al., 2020) function in Python.

### Microbial Community Analysis

Samples for 16S rRNA analysis were taken at three time points. Six mL of each incubation replicate sample was set aside for immediate DNA extraction after sampling. Sampling was performed 9, 18, and 29 days after the start of the experiment. Three mL of wet sediment was used to determine the microbial composition of UML sediment. DNA extractions were performed using the PowerSoil (Qiagen) DNA extraction kit following the manufacturer’s instructions for “wet soil.” Accordingly, the samples were centrifuged at 10,000 g in 1.5 mL microcentrifuge tubes to remove excess water, adding 1.25 mL at a time and then discarding supernatant before adding another 1.25 mL of sample and centrifuging with discarding supernatant again until the entire sample (6 mL for incubations and 3 mL for UML sediment) was processed.

Amplicon libraries were prepared using methods described in Preheim et al. (2013) and sequenced using Illumina MiSeq 2 × 250 paired-end at the BioMicroCenter (Massachusetts Institute of Technology, Cambridge, MA, United States). Universal primers U515F (PE16S_V4_U515_F ACACGACGCTCTTCCGATCTYRYRGTGCCAGCMGCCGCG GTAA) and E786R (PE16S_V4_E786_RCGGCATTCCTGCT GAACCGCTCTTCCGATCTGGACTACHVGGGTWTCTAAT) were used (Preheim et al., 2013). Primers were removed from forward and reverse reads using a python script, allowing one mismatch and discarding reads without the primer. The reads were then quality filtered and truncated to a common length, allowing up to two expected errors and truncating to 200 bases for the forward reads and 185 for the reverse. DADA2 (Callahan et al., 2016) was used to infer the Amplicon Sequence Variants (ASVs) from the raw reads. Pseudo-pooling was performed by running DADA2 twice and using all identified ASVs from the first run as prior sequences in the second, as described on the pooling documentation on the DADA2 website. This reduces the risk of false negatives with DADA2 where sequences with only one or two reads that are close to a more abundant sequence are erroneously corrected. ASVs were assigned taxonomy using the DADA2 assignTaxonomy function and the RDP database (Cole et al., 2014).

All statistical analyses on 16S rRNA ASV data were carried out in R (version 3.5.1) (R Core Team, 2018). Analysis of the microbial communities and initial plotting were performed using the Phyloseq package in R (McMurdie and Holmes, 2013) which borrows many functions from Vegan (Oksanen et al., 2019). Plots were made using ggplot2 (Wickham, 2016). Pairwise permutational multivariate analysis of variance (PERMANOVA) comparisons were made using the RVAideMemoire package (Hervé, 2020). DESeq2 was used on count data for normalization and differential abundance analysis using default analysis parameters (Love et al., 2014).

## Results

### Thermodynamic Calculations

Thermodynamic calculations were used to determine the relative yields of iron reduction of hematite, goethite, and ferrihydrite reduction, as well as methanogenesis. Under standard state conditions, magnetite reduction yields the most energy, followed by ferrihydrite, goethite, and hematite reduction. Methanogenesis yields the least amount of energy (Table 1). However, when considering importance for microbial metabolisms, these reactions must be considered under environmentally relevant chemical conditions. As such, thermodynamic calculations for acetate-consuming iron oxide reduction and acetoclastic methanogenesis were conducted over a range of product activities at representative environmental conditions (Table 1 and Supplementary Figure 3). As expected, all iron-reducing reactions have increasing ΔG_rxn_ (decreasing favorability) with increasing [Fe^2+^_(aq)_]. Acetoclastic methanogenesis also has an increasing ΔG_rxn_ with increasing [CH_4(aq)_]. Changing acetate activities by orders of magnitude (e.g., approximately equivalent to 1 μM to 10 mM concentrations) has no effect on the relative favorabilities of each reaction (Table 1 and Supplementary Figure 3).

When considering acetate as the sole electron donor, thermodynamic calculations predict that ferrihydrite is the most energetically favorable iron oxide electron acceptor followed by goethite and hematite, as shown previously (Bonneville et al., 2004; Bethke et al., 2011). The reaction stoichiometry of iron reduction is the same for these three minerals, producing 8 mol Fe per mol acetate oxidized. This highlights that the favorability of iron reduction is controlled by Fe^2+^ activity in a similar manner for each mineral. Therefore, the relative energy yield of reduction of these iron oxides is always ferrihydrite reduction, followed by goethite reduction, and then hematite reduction, with energy yield decreasing as [Fe^2+^_(aq)_] increases.

At activities of Fe^2+^ higher than ∼10 uM, ferrihydrite reduction is always more favorable than methanogenesis, followed by goethite reduction and hematite reduction, with magnetite reduction being least favorable. An order of magnitude change in[(Fe^2+^_(aq)_] leads to a much greater change in ΔG_rxn_ than an order of magnitude increase in acetate or decrease in methane concentrations. The ΔG_rxn_ for acetotrophic iron reduction increases by +45.6 kJ/mol acetate oxidized with a one order of magnitude increase in iron, whereas the ΔG_rxn_ only increases by +5.7 kJ/mol acetate oxidized with a one order of magnitude decrease in acetate activity. The ΔG_rxn_ for methanogenesis increases by +5.7 kJ/mol acetate for an order of magnitude increase in [CH_4(aq)_] or an order of magnitude decrease in acetate activity.

### Methane Production and Iron Speciation

**FIGURE 2:**
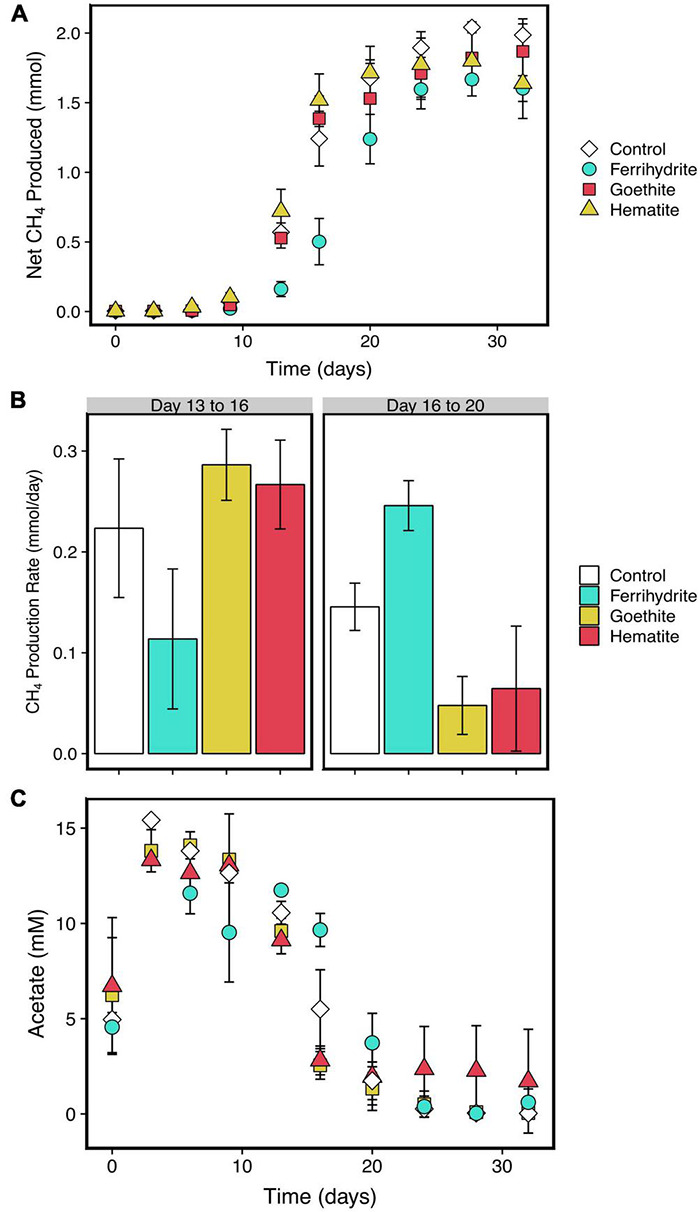
Net methane production in the presence of each mineral or Fe-free control **(A)** and average daily rates of methane production from Days 13 to 16 and Days 16 to 20, which correspond to maximum measured methane production rates for hematite, goethite, and control; and ferrihydrite incubations, respectively **(B)**. Acetate concentrations over time are shown in **(C)**. Error bars in both panels are standard deviations of three replicates. Points without error bars have standard deviations smaller than the symbols.

**FIGURE 3:**
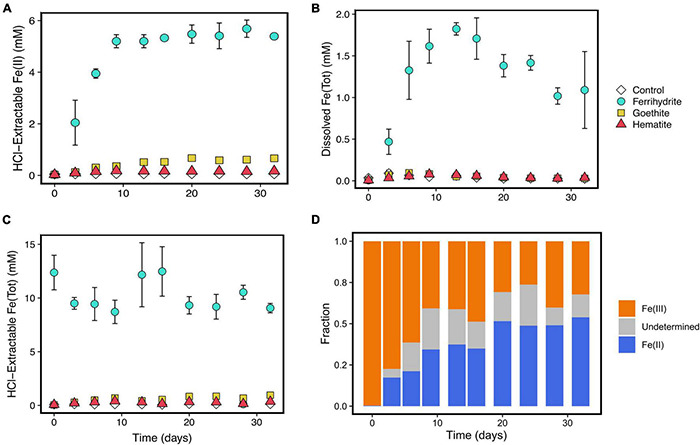
Fe speciation over the course of incubation experiments including, hydrochloric acid-extractable Fe(II) **(A)**, aqueous Fe **(B)**, total HCl-extractable Fe **(C)**, and HCl-extractable solid speciation **(D)**. Error bars represent standard deviations of three replicates. Points without error bars have standard deviations smaller than the symbols. Error bars are left out of panel **(D)** for viewing clarity; values and error are shown in Supplementary Table 3. Because aqueous Fe speciation was not determined, a portion of the solid HCl-extractable phase could not be determined because the total HCl-extractable Fe concentrations also include aqueous Fe.

Methane was measured to determine the timing of the onset of methanogenesis and rates of methane production. In all experiments, the partial pressure of headspace methane increased throughout the length of the 30 days following a S-growth curve-like shape (Figure 2A). Under all reaction conditions, total methane produced was about 2 mmol. The first substantial increase in methane production in all incubations occurred between days 9 and 13, which coincides with a termination of HCl-extractable and aqueous Fe(II) production in ferrihydrite incubations (Figure 3).

Maximum methane production rates were similar across all experiments at about 0.2 to 0.3 mmol per day (Figure 2B); however, there was a longer lag time in ferrihydrite experiments before the maximum methane production rate was reached. The maximum measured rate of methane production was reached between days 13 and 16 in goethite, hematite, and control experiments, whereas methane production had only reached about 0.1 mmol per day in ferrihydrite experiments. In contrast, the methane production rate in ferrihydrite experiments increased to about 0.25 mmol per day on average between days 16 to 19, while the rate decreased to an average of 0.15 mmol per day or less under all other experimental conditions. Previous tracer experiments show that essentially all methane was produced from the carboxyl carbon from acetate (see Supplementary Figure 5 for more information).

The relative consumption rate of acetate was similar to that of methane production in all samples (Figure 2C). Acetate concentrations initially increased to 15 mM by day 3, and then decreased over time until acetate concentrations were indistinguishable from 0. Ferrihydrite reduction led to a relatively small amount of acetate consumed before the onset of methanogenesis, despite substantial iron reduction earlier in the experiment. Like methane production, the maximum acetate consumption rate was reached between days 13 and 16 under all experimental conditions except for the ferrihydrite incubations, which reached a maximum between days 16 and 20.

Iron concentrations and speciation were measured over time to understand iron reduction. HCl-extractable Fe(II) concentrations increased the most in ferrihydrite incubations, despite hematite and goethite experiments having stoichiometrically equivalent amounts of oxidized iron added at the onset of the experiments (Figure 3A). HCl-extractable Fe(II) concentrations in ferrihydrite cultures reached a maximum concentration of about 6 mM after 10 days. Meanwhile, dissolved Fe reached concentrations of about 2 mM (Figure 3B), and total HCl-extractable Fe stayed constant at about 10 mM throughout the 29 days (Figure 3C). In contrast, HCl-extractable Fe(II)/Fe(III) and dissolved Fe concentrations remained much lower throughout the course of the experiments in the hematite, goethite, and control cultures. Less than 1 mM of HCl-extractable Fe(II) was produced in each of these incubations.

The solid phase in the ferrihydrite incubations also changed over time (Figure 3D). The acid-extractable solid Fe started as exclusively Fe(III) but incorporated Fe(II) over the course of the experiment. This rate of incorporation slowed after 9 days and leveled off at a minimum of about 30–50% Fe(II) incorporation. Additionally, over the course of the experiment, there was a color change from reddish-brown to dark brown/black in the ferrihydrite experiments. All other experiments stayed consistent in color, with hematite incubations remaining red and goethite incubations remaining yellowish-orange.

### Overview of Microbial Diversity and Trends

16S rRNA amplicon sequencing was used to determine the composition of the microbial communities in response to the different iron oxide amendments within the incubations over time. DNA extractions were done at 9, 18, and 29 days into the experiments. These experimental samples were compared to samples collected from Upper Mystic Lake (UML) sediment, which was used as the inoculum for these experiments. After processing, there were 6,388,081 sequences in total in the 38 samples. These sequences were associated with 7720 ASVs. The UML sediment had a much higher diversity than the incubation samples; the UML samples had on average 4075 ASVs (± standard deviation of 133 ASVs) while the incubation samples contained 1060 ASVs on average (± standard deviation of 180 ASVs) (Supplementary Figure 5A), with little deviation between incubation conditions, and all samples had similar species evenness (Supplementary Figure 5B).

At the phylum level (Supplementary Figures 6–8), all incubation samples were dominated by Bacteroidetes and Firmicutes. The abundance of Euryarcheota, which contains most anaerobic methanogens, was lower for all incubation samples than in the original UML sediment at the day 9 time point. However, Euryarcheota increased to at least 10% relative abundance in all samples throughout the course of the incubation. In contrast, the initial UML sediment was dominated by predominantly Proteobacteria, with some Bacteroidetes, Chloroflexi, and Euryarchoeta. Over 25% of the relative abundance of UML sediment consisted of ASVs that could not be classified at the phylum level.

**FIGURE 4:**
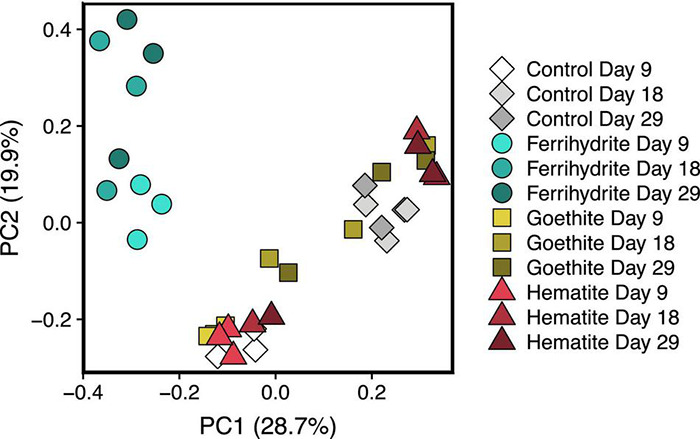
PCoA plot of microbial communities based on ASV relative abundance data. Each time point for each experimental condition has 3 replicates represented separately.

Principle component analysis (PCoA) on the Bray-Curtis dissimilarity matrix of the experimental samples at the ASV level consisted of two distinct clusters (Figure 4). One cluster contained the ferrihydrite samples from all three time points whereas the other cluster contained the hematite, goethite, and Fe oxide-free control samples at all time points. The two clusters became more dissimilar over the 20 days between the first and last 16S rRNA time point. Similar clusters also form when taxa are grouped at the genus level (Supplementary Figure 9). Pairwise PERMANOVA comparisons, used to distinguish significantly different microbial communities, agreed with visual trends in the PCoA plot (Supplementary Table 2); pairwise comparisons of the ferrihydrite group of incubation samples compared to hematite, goethite, and control groups all had *p*-values of < 0.01. In contrast, all other pairwise group comparisons had *p*-values of > 0.01, and all except goethite-ferrihydrite had *p*-values > 0.05.

### Key Players in Methanogenesis and Iron Reduction

**FIGURE 5:**
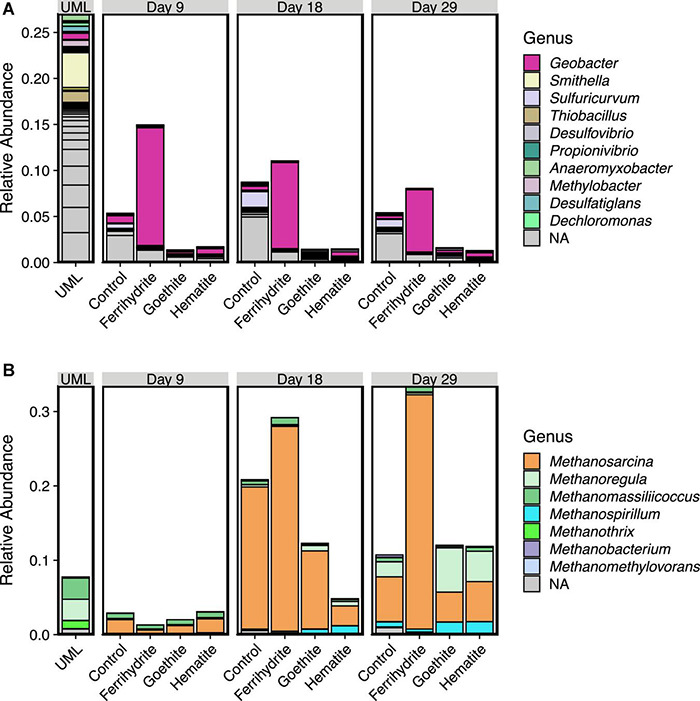
Relative abundance plots of 16s-rRNA data by genera for the phyla Proteobacteria **(A)** and Euryarcheota **(B)**. Genera are listed in order from most to least abundant (sum total across all samples) and only genera with a mean relative abundance of 0.001 or greater are included in the legend. Gray bars represent ASVs that could not be classified at the genus level, with breaks within gray bars reprsenting multiple ASVs. Each bar is the average of three experimental replicates. Upper Mystic Lake (UML) sediment data is shown for comparison.

Relative abundances of genera within the phyla Proteobacteria and Euryarcheota were explored in more detail to provide insight into the microbes carrying out Fe(III) oxide reduction and methanogenesis, respectively. Figure 5 shows the relative abundances of Proteobacteria (Figure 5A) and Euryarcheota (Figure 5B) at the genus level. The relative abundance of *Geobact*er substantially increased above the initial UML sediment relative abundance in ferrihydrite samples. After a large initial increase in *Geobacter* abundance between initial UML sediment and ferrihydrite incubations at the day 9 time point, the relative abundance of *Geobacter* decreased in ferrihydrite incubations until day 29. In all incubations without ferrihydrite, the relative abundance of *Geobacter* was similar to the UML sediment.

At day 9, ferrihydrite incubations had the lowest relative abundance of methanogens compared to hematite, goethite, and Fe oxide-free control samples. The relative abundance of Euryarcheota increased substantially across all samples from ≤ 3% at day 9 to over 10% on average by day 18. Interestingly, at day 18 and day 29, ferrihydrite incubations contained a higher relative abundance of Euryarchaeota than all other experimental samples. In all incubations, Euryarcheota consisted of predominantly *Methanosarcina* after 18 days with an ingrowth of *Methanoregula* in all incubation samples except for the ferrihydrite incubations after 29 days. In contrast, the most prominent methanogens in the initial UML sediment were *Methanomassillicoccus*, *Methanoregula*, and *Methanothrix*, which all decreased in relative abundance from a total of about 7% in UML sediment to <1% by day 9 in every incubation sample.

### ASV-Level Differences Between Experimental Conditions

**FIGURE 6:**
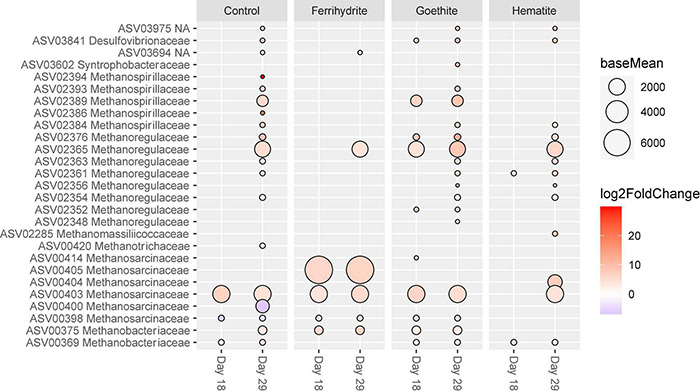
ASVs classified as Proteobacteria and Euryarchaeota with significant (*p* < 0.01) log-fold changes (*p* < 0.01) over time. Each sample was compared its corresponding “Day 9” sample (e.g., Control Day 29 vs. Control Day 9). The size of the circle corresponds to the normalized mean counts of each ASV, while the color corresponds to the magnitude of the log-fold change.

DESeq2 was used to determine which ASVs were significantly differently abundant over time and across experimental conditions. First, all mineral and control incubations were analyzed based on which ASVs significantly changed over time in order to contextualize comparisons across experimental conditions. For each experimental condition, sequencing data from the two later time points (Day 18 and Day 29) were compared to the corresponding data at Day 9. Overall, ferrihydrite-amended communities were most stable across the three time points, with only 25 ASVs that significantly changed between day 9 and day 29, compared with ∼100 ASVs that changed significantly under all other experimental conditions. ASVs that were significantly different between Day 9 and later time points that belong to the phyla Proteobacteria and Euryarchaeota are shown in Figure 6.

The changes in relative abundance between apparent iron reducers and methanogens occurred at different times throughout the course of the incubation experiments. There were no significantly different ASVs belonging to the family Geobacteraceae from days 18 and 29 compared to day 9, indicating that Geobacteraceae abundances stayed generally consistent across all three time points. In contrast, some of the most abundant Methanosarcinaceae ASVs showed significant differences between day 9 and days 18 and 29. For example, ASV00403 abundance significantly increased over time across all experimental conditions. However, ASV00405, which on average was the most abundant ASV of methanogen, only increased significantly after day 9 with the addition of ferrihydrite. Most methanogen ASVs with significant changes increased by day 29, except for two less abundant Methanosarcinaceae ASVs (ASV00398 and ASV00400) that decreased in relative abundance.

Differential abundance data were also examined across conditions by comparing each mineral incubation to the control incubations at each time point in order to determine what was driving the differences between PCoA clusters (Figure 4). In accordance with the PCoA analysis, ferrihydrite incubations had the highest number of ASVs that were significantly different from the control at each time point (Supplementary Figure 10). The number of ASVs different from the control at each time point increased in ferrihydrite samples from 73 at day 9 to 143 at day 29.

**FIGURE 7:**
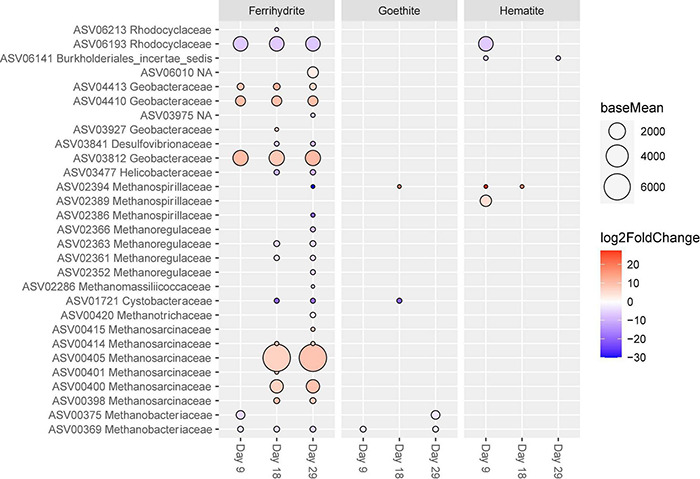
ASVs classified as Proteobacteria and Euryarchaeota with significant (p < 0.01) log-fold changes from control experiments. Each mineral addition is compared to its time-corresponding control sample (e.g., “Ferrihydrite Day 18” vs. “Control Day 18”). The size of the circle corresponds to the normalized mean counts of each ASV, while the color corresponds to the magnitude of the log-fold change.

Proteobacteria and Euryarchaeota ASVs that were significantly differently abundant compared to control incubations are shown in Figure 7. Four ASVs classified as Geobacteraceae (ASV04423, ASV04410, ASV 03927, and ASV03812) were significantly more abundant in ferrihydrite experiments than controls, and this increased abundance was consistent across all three time points. There were no Geobacteraceae ASVs that were significantly less abundant in ferrihydrite incubations than control incubations, and there were no Geobacteraceae ASVs that were significantly different in hematite and goethite incubations from control incubations.

Methanogen relative abundances generally only showed significant differences between ferrihydrite and control experiments, and these differences mostly appeared after Day 9. The number of methanogen ASVs that were significantly different from control experiments increased throughout the course of the incubation from two, both belonging to the family Methanobacteriacaeae and at lower relative abundance than controls, at day 9 up to a total of seventeen at day 29. Specifically, the presence of ferrihydrite specifically enriched for ASVs in the Methanosarcinaceae family, with one ASV (ASV00405) being especially abundant in ferrihydrite relative to all other experimental conditions, while all other methanogen families (including Methanobaceriaceae and Methanospirillaceae) were found in significantly lower abundance relative to control experiments. In contrast, three or fewer ASVs of methanogens were significantly different between goethite or hematite and control experiments at every time point.

Heat map plots of the top 100 ASVs across all samples (Supplementary Figure 11), using PCoA as the ordination method on Bray-Curtis dissimilarity matrices of relative abundance data, provide nice agreement with differential abundance analysis for iron reducers and methanogens. Notably, there is a cluster of 17 ASVs that all appeared to be highly abundant in ferrihydrite incubations across all time points and less abundant in hematite, goethite, and control incubations. This cluster contains three methanogen ASVs, including *Methanosarcina* ASV00405, and four ASVs in the Geobacteraceae family, including ASV03812.

## Discussion

While incubations with hematite, goethite, and ferrihydrite had equivalent amounts of Fe(III) added, only ferrihydrite was substantially reduced over the course of these experiments. Iron reduction proceeded readily in the presence of ferrihydrite using acetate as an electron donor, reaching mM concentrations of acid-extractable Fe(II) and aqueous Fe. However, acid-extractable Fe(II) concentrations remained about an order of magnitude lower in goethite and hematite incubations, indicating a much smaller extent of Fe(III) reduction than in ferrihydrite experiments. These trends in relative reduction amounts agree well with previous investigations of Fe(III) reduction in the presence of different Fe oxide minerals (Lovley and Phillips, 1988; Cutting et al., 2009).

Iron reduction in ferrihydrite incubations corresponded with an increase in *Geobacter* abundance relative to all other incubation conditions, with four ASVs being significantly higher in relative abundance compared to control incubations across all three time points. The relative abundance of *Geobacter* reached a maximum by day 9 of the incubation experiments, which is also approximately the point when iron reduction stopped. The relative abundance then decreased over time; however, this decrease was not statistically significant for any individual ASV of *Geobacter.*

Thermodynamic predictions of iron reduction agree well with incubation experiments. Since these experiments were done in artificial freshwater (ionic strength ∼0.02, see “Materials and Methods” for background solution composition), the concentrations of aqueous constituents are roughly equal to activities (especially when considered on a log scale as in Supplementary Figure 3). Iron(III) reduction proceeded readily in the presence of ferrihydrite using acetate as an electron donor, reaching mM concentrations of acid-extractable Fe(II) and aqueous Fe. However, acid-extractable Fe(II) concentrations remained about an order of magnitude lower in goethite and hematite incubations, indicating a much smaller extent of Fe(III) reduction than in ferrihydrite experiments. These trends in relative reduction amounts agree well with previous investigations of Fe(III) reduction in the presence of different Fe oxide minerals (Lovley and Phillips, 1988; Cutting et al., 2009).

Thermodynamic predictions also explain how microbial ferrihydrite reduction outcompetes methanogenesis over a wider range of environmental conditions than hematite and goethite reduction. For conditions where Fe^2+^_(aq)_ activities are as high as 10^–2^, ferrihydrite reduction is always more favorable than methanogenesis. At very low Fe^2+^_(aq)_ activities (e.g., less than 10^–7^), hematite and goethite reduction are also more favorable than methanogenesis. However, due to reaction stoichiometry; 8 moles of Fe^2+^ are produced for every 1 mole of acetate consumed while only 1 mole of CH_4_ is produced per mole acetate consumed (Table 1). Thus, the ΔG_rxn_ of acetoclastic methanogenesis increases much less with increasing methane activities compared to the ΔG_rxn_ of iron reduction with equivalent increases in Fe^2+^_(aq)_ activities. This results in methanogenesis being favorable over a wider range of methane activities compared to hematite and goethite reduction over a range of Fe^2+^_(aq)_ activities. Therefore, methanogenesis is expected to dominate over iron reduction across a range of environmentally relevant conditions when hematite and goethite are the available electron acceptors, but ferrihydrite reduction remains more favorable than methanogenesis even at high Fe^2+^_(aq)_ activities.

Acetate concentrations also varied over the course of the experiment. Initially, acetate concentrations increased to ∼15 mM. This may be due to fermentation of organic matter from the Upper Mystic Lake sediment used as an initial microbial community source. ASVs from genera of bacteria with members known to be capable of producing acetate as a fermentation product, including Macellibacteroides and Romboutsia (Jabari et al., 2012; Wang et al., 2015), were highly abundant by day 9 (Supplementary Figures 7, 8). After day 9, when concentrations stopped increasing, relative acetate consumption rates were similar to relative methane production rates; acetate consumption in the presence of ferrihydrite was slower than in the presence of hematite and goethite. Although acetate oxidation by Fe reducers likely also contributed to acetate consumption in the presence of ferrihydrite before the onset of methane production, this amount would be relatively small due to the stoichiometry of the Fe reduction and methanogenesis reactions. Approximately 1.5-2 mmoles of ferrihydrite was reduced to Fe(II) (6 mM in < 200 mL solution with liquid sample removal) and 2 mmoles of methane were produced. As mentioned previously, 8 moles of Fe(III) reduction are needed to oxidize 1 mole of acetate while 1 mole of methane production consumes 1 mole of acetate, meaning only approximately 1/8 the amount of acetate consumed went toward ferrihydrite reduction compared to methanogenesis.

However, despite thermodynamic predictions, ultimately, iron reduction in ferrihydrite incubations only influenced methane production in the beginning of the incubation timeframe until approximately day 9 (Figure 2). The time taken to reach the maximum methane production rate was delayed relative to incubations with crystalline iron oxides but methanogenesis was not completely inhibited in the presence of ferrihydrite. Despite this delay, the maximum rates of methane production and final methane concentrations were comparable within error between all iron oxide additions and control incubations. This is interesting because as mentioned previously, thermodynamic calculations show that ferrihydrite reduction should still be more favorable than methanogenesis, even at 1 mM aqueous Fe(II) concentrations (Supplementary Figure 4). Despite iron reduction being more thermodynamically favorable, methanogens were still able to grow and produce methane. Therefore, thermodynamic predictions alone are not enough to explain interactions between iron-reducing bacteria and methanogens.

The termination of iron reduction at about day 9 may be explained by the transformation of ferrihydrite into other more crystalline iron oxides. Ferrihydrite is a metastable phase that converts into other minerals over time like magnetite, goethite, and lepidocrocite depending on solution Fe(II) concentrations (Hansel et al., 2005). These transformations occur on the order of hours to days when in solution with aqueous Fe(II) (Hansel et al., 2003). As previously mentioned, the ferrihydrite incubations underwent a color change from translucent reddish brown to opaque black/dark brown, suggesting a phase change. Additionally, HCl-extractable Fe(II) concentrations (Figure 3) were about three times higher than total dissolved Fe concentrations (Supplementary Figure 3A) by day 9, indicating that the majority of the produced Fe(II) had been adsorbed and/or incorporated into the ferrihydrite. At relatively high Fe^2+^_(aq)_ concentrations, such as those seen in the ferrihyrite incubations in this study, both goethite and magnetite reduction are less favorable than methanogenesis (Table 1 and Supplementary Figure 3). Lepidocrocite has the same chemical formula as goethite and a very similar ΔG°_f_ (Cornell and Schwertmann, 2003), so thermodynamic calculations would lead to almost identical favorabilities of Fe(III) reduction across reactant and product activity space. Thus, ferrihydrite transformation into magnetite, goethite, and/or lepidocrocite could account for the observed shift to methanogenesis from Fe reduction despite ferrihydrite reduction still being favorable under experimental conditions. However, total amounts of HCl-extractable Fe in ferrihydrite incubations stayed consistent at approximately 10 mM (Figure 3C), which means that if these minerals formed, they would have been poorly crystalline enough to still dissolve during the acid extraction procedure.

It is also possible that surface passivation by formation of Fe(II) precipitates contributed to the eventual outcompeting of Fe-reducers by methanogens in ferrihydrite incubations. Roden and Zachara (1996) found that formation of an Fe(II) surface phase in experiments with *Shewanella alga* limited iron reduction compared to initial Fe reduction rates. Additional experiments by Roden and Urrutia (2002) showed similar results with *Geobacter metallireducens* and their work also suggests that Fe(II) sorption onto iron-reducing bacteria may further inhibit their ability to carry out iron reduction. Friese et al. (2021) observed methanogenesis dominating over iron reduction in sediments in ferruginous Lake Towuti, Indonesia despite the presence of reactive iron(III) phases and attribute this to surface passivation of reactive iron. Our observed increase in Fe(II) content of the ferrihydrite (Figure 3D) over time is also consistent with surface passivation, which may have contributed to our observed results showing that methanogens can outcompete iron-reducing bacteria on longer timescales in the presence of ferrihydrite.

Despite similar methane production rates and extents, there were marked differences in microbial community development in the presence of ferrihydrite compared to controls with no added iron that did not arise with the addition of hematite or goethite. The decreased diversity of the experimental samples relative to the microbial community in the original UML sediment indicates that the experimental conditions selected for specific microbes. Two distinct clusters (Figure 5) corresponding to incubations with ferrihydrite and without ferrihydrite (hematite, goethite, and Fe-free control) formed by day 9, and the distance between these clusters increased throughout the course of the incubation experiments. In contrast, the presence of crystalline oxides hematite and goethite, which did not change the rate or time to onset of methane production relative to an Fe-free control, also did not have a distinct effect on the resulting microbial communities.

The influence of ferrihydrite on the methanogen community became especially apparent between day 9 and day 29 (Figure 4). The presence of ferrihydrite enriched for multiple *Methanosarcina* ASVs relative to control experiments by day 18 and these ASVs continued to be at high relative abundance at day 29 (Figure 7). Additionally, even though all samples had an overall high relative abundance of *Methanosarcina*, ferrihydrite incubations enriched for a unique ASV of *Methanosarcina* (ASV00405) that was not as highly abundant in the control, goethite, and hematite samples. It is unclear if this ASV is simply more tolerant to high Fe(II) concentrations, or if some other unaccounted for process is occurring only in ferrihydrite samples. At the genus level, *Methanosarcina* includes both acetoclastic (Smith and Mah, 1978; Jetten et al., 1992) and hydrogenotrophic (Ferguson and Mah, 1983) methanogens, but the methanogens in our study appeared to be performing acetoclastic methanogenesis (see Supplementary Figure 4). However, ferrihydrite incubations had a decreased relative abundance of all ASVs significantly different from controls that belonged to other methanogen families, including known hydrogenotrophic families such as Methanobacteriaceae (Oren, 2014a) and Methanoregulaceae (Bräuer et al., 2011; Oren, 2014b), and these ASVs were observed to be at significantly lower abundance only at day 29.

Consistent with our results, a number of other laboratory incubation studies have also observed enrichment of *Methanosarcina*, often with *Geobacter*, in the presence of iron oxides, or other (semi)conductive minerals, and acetate. These include experiments with paddy soil with additions of both ferrihydrite and magnetite (Zhuang et al., 2015b), as well as incubations with lake sediments with added granular activated carbon (GAC) (Rotaru et al., 2018a) and crystalline iron oxides (Aromokeye et al., 2018). However, in the field, *Methanothrix* is shown to be the dominant methanogen in the iron-rich sediments of Lake La Cruz (Rotaru et al., 2018b), and the most abundant non-hydrogenotrophic methanogen in the water column of ferruginous Lake Matano (Crowe et al., 2011). This is also the case for our study, where in original UML sediments, *Methanothrix* was the most abundant genus of acetoclastic methanogens behind hydrogenotrophic methanogens *Methanomassiliicoccus* and *Methanoregula*. Additional work is needed to understand the reason for this discrepancy between laboratory enrichments and field observations.

The results of this work contrast with a study by Roden et al., in which the presence of poorly crystalline iron oxides prohibited methanogenesis over the length of a 40 day experiment (Roden, 2003). In that study, incubation acetate concentrations were an order of magnitude lower than used here and this concentration was specifically chosen to make acetate a limiting factor, which may account for the difference (Roden, 2003). Other studies using higher acetate concentrations comparable to those used in our work have shown similar results to this study, i.e., delayed methanogenesis with the addition of ferrihydrite in *Geobacter/Methanosarcina* co-cultures (Tang et al., 2016) and soil-inoculated experiments (Kato et al., 2012; Zhuang et al., 2015b). It is therefore possible that the complete suppression of methanogenesis in the presence of ferrihydrite is dependent on the amount of acetate remaining in solution following Fe(III) reduction. When acetate is not consumed completely in the process of iron reduction, methanogens can eventually out-compete iron-reducers once iron reduction is no longer feasible, possibly due to the conversion of ferrihydrite to other minerals like magnetite and goethite or by surface passivation as a result of Fe(II) mineral precipitation as discussed previously. Yet, other environmental factors, including the initial form of ferrihydrite, transformation to other iron oxides, and solution conditions (e.g., pH) would also influence the extent of methane production or suppression.

This study also does not take into account potential cooperative processes between iron reducing bacteria and methanogens, like direct interspecies electron transfer (DIET), that have been demonstrated to occur in the presence of hematite and magnetite, among other (semi)conductive materials (Rotaru et al., 2014, 2018a; Zhuang et al., 2015a; Martins et al., 2018). DIET involves iron-reducers transferring electrons resulting from organic carbon oxidation to methanogens using (semi)conductive materials as a conduit. There have also been studies showing that magnetite can accelerate acetolactic methanogenesis directly (Fu et al., 2019; Inaba et al., 2019). While our work using ^13^C-labeled methane in a previous experiment (Supplementary Figure 4) did not show any evidence of DIET, it is possible that DIET may be selected for when using a different sediment or soil as an inoculum. It is also possible that a higher temperature could have influenced DIET, as a temperature of 30°C stimulated methanogenesis to a greater extent in the presence of hematite and magnetite in Aromokeye et al. (2018) compared to experiments at 10°C. Additionally, many studies that have observed DIET in the presence crystalline iron oxides have used substrates that could not be directly utilized by methanogens, including glucose (Aromokeye et al., 2018) and benzoate (Aromokeye et al., 2020b); addition of a substrate unusable for methanogenesis may also select for organisms capable of DIET.

Moreover, methanogens have been shown to directly contribute to iron cycling. Some methanogens have even been able to reduce iron(III) in laboratory studies (Sivan et al., 2016), and methanogens reducing Fe(III) may also increase the rate of iron reduction relative to methanogenesis (Van Bodegom et al., 2004). It is possible that the methanogen ASVs selected for in ferrihydrite experiments are also capable of reducing iron. Anaerobic oxidation of methane (AOM) coupled to reduction of iron (Sivan et al., 2014; Bar-Or et al., 2017), as well as other electron acceptors (Treude et al., 2003), can also influence net methane emissions. However, generally, observed Fe-AOM rates have been much slower than the methane production rates reported in this study (Beal et al., 2009; Ettwig et al., 2016). For example, Aromokeye et al. (2020a) observed incubation Fe-AOM rates of about 0.1 μM per day while Ettwig et al. (2016) observed about 1.5 μmols CO_2_ formation per L of enrichment culture in Fe-AOM enrichment experiments in the presence of ferrihydrite, compared to net methane production rates of up to 1.5 mmol per day per L starting solution in this work.

Additionally, more information about environmental conditions must be taken into account when determining applicability of our experiments at specific field conditions. For example, naturally formed iron oxide minerals, like goethite, can have a higher surface area than laboratory-synthesized iron oxides, which will influence their susceptibility to iron reduction (Roden, 2003). Other factors, such as pH (Bethke et al., 2011; Jin and Kirk, 2018) and ligands like organic acids (Schwertmann, 1991), can have an influence on the reactivity and solubility of iron oxides, which can affect the extent of reduction of iron oxides and subsequently methanogenesis (Miller et al., 2015; Marquart et al., 2019).

Varying crystallinities of iron oxides have been invoked previously as an explanation for overlapping zones of sulfate and iron reduction (Postma and Jakobsen, 1996), and this is also likely applicable for methanogenesis and iron reduction. One study looking at Baltic Sea sediments demonstrated evidence for Fe(III) reduction outcompeting methanogenesis in the zone of methanic sediments with poorly crystalline iron oxides, but saw methanogenesis co-occurring with crystalline iron oxide reduction in a deeper zone (Egger et al., 2017). Additionally tropical soils are also large natural sources of methane (Oertel et al., 2016) and often consist of Oxisols and Ultisols rich in hematite and goethite (Wiriyakitnateekul et al., 2007); however, to the best of our knowledge, the relationship between iron oxide structure and methane production in tropical soils has not been studied directly, and previous research in these soils has mainly focused on poorly crystalline iron oxides (Teh et al., 2008).

This work demonstrates the influence of iron oxide structure in determining the rate and extent of iron oxide reduction compared to methanogenesis systems with non-limiting concentrations of acetate. More well-crystalline iron oxides hematite and goethite had little effect on methane production or microbial community development throughout the course of the experiment. While the presence of ferrihydrite led to an initial surge of iron reduction throughout the first 9 days of the experiment, ferrihydrite likely transformed into more crystalline iron oxides like magnetite and goethite or underwent surface passivation by Fe(II) minerals over time. This eventually allowed methanogenesis to dominate, although the rate of methane production was slowed until after iron reduction stopped. By the end of the incubation experiments, there was no distinguishable difference in the maximum rate or total extent of methane production under any of the experimental conditions.

The fact that acetate was not limiting relative to iron also likely played a role in the ability of methanogens to become more relatively abundant than iron reducers even in the presence of ferrihydrite. As mentioned previously, addition of excess acetate was found to increase methane production rates by a factor of more than fifty while only doubling iron reduction rates in tropical rain forest soil incubations (Teh et al., 2008). It has been also previously observed that iron reducers can deplete acetate and H_2_ to lower concentrations than sulfate reducers and methanogens in freshwater sediments, and that at high substrate concentrations all three processes could occur simultaneously (Lovley and Phillips, 1987). Additional work directly comparing incubations with high and low acetate concentrations could provide more insight into the role of electron donor availability on competition between iron reduction and methanogenesis.

The results of this research suggest that the duration and/or frequency of redox cycles, especially in environments with high concentrations of organic acids, like acetate, is important in determining the relative balance of iron reduction compared to methanogenesis in the environment. Tropical soils specifically can undergo redox oscillations due to periods of high water content as a result of intense rainfall events (Liptzin et al., 2010), which can have an influence on iron oxide mineralogy. The crystallinity of iron oxides in soils is linked to both initial mineralogy and occurrence of redox oscillations (Thompson et al., 2006), and frequent redox oscillations have been found to increase iron reduction rates in soils (Ginn et al., 2017; Barcellos et al., 2018). In this work, we found that the presence of ferrihydrite alone was not enough to change long-term methane production in the absence of carbon limitation, as ferrihydrite likely transformed into more well-crystalline minerals or underwent surface passivation over time before the carbon was completely utilized. The redox fluctuations that affect iron reduction by influencing iron oxide crystallinity therefore also probably affect methanogenesis.

The research has important implications for global methane cycling in environments with iron oxides, especially those that are not carbon-limited, on the timescales explored in this study. Because ferrihydrite transforms into more crystalline minerals like goethite and magnetite on a time scale of weeks under iron-reducing conditions, many water-saturated soil and sedimentary systems have crystalline iron oxides as the most highly abundant form of iron oxides at sub/anoxic depths (Wiriyakitnateekul et al., 2007; Kocar and Fendorf, 2009; Stuckey et al., 2016). Many of these soil/sedimentary systems may have been previously overlooked as potential sources of methane due to their high iron oxide contents. This has implications for global methane models, which typically only include soils as sinks of methane (Kirschke et al., 2013). Being able to characterize individual soils as methane sources or sinks based on mineralogy and carbon substrate availability will allow for more accurate predictions of global methane emissions and consequently future climate change.

## Data Availability Statement

The datasets presented in this study can be found in online repositories. The names of the repository/repositories and accession number(s) can be found below: https://www.ncbi.nlm.nih.gov/sra and BioProject PRJNA725260, https://github.com/hjgadol/Gadol_FeCH4, Geochemical Data.

## Author Contributions

HG and BK designed the study. HG carried out the experiments, performed the geochemical analyses and DNA extractions, and wrote the manuscript. JE and HG contributed to the metagenomic data analysis. BK and JE contributed to the writing of and revised the manuscript. All authors contributed to the creation of the manuscript.

## Conflict of Interest

BK was employed by Exponent, Inc. The remaining authors declare that the research was conducted in the absence of any commercial or financial relationships that could be construed as a potential conflict of interest.

## Publisher’s Note

All claims expressed in this article are solely those of the authors and do not necessarily represent those of their affiliated organizations, or those of the publisher, the editors and the reviewers. Any product that may be evaluated in this article, or claim that may be made by its manufacturer, is not guaranteed or endorsed by the publisher.
