# Supplementary material for: Methanogen Productivity and Microbial Community Composition Varies With Iron Oxide Mineralogy
Source: Front Microbiol. 2022 Feb 18;12:705501. doi: 10.3389/fmicb.2021.705501 (PMC8894893; doi:10.3389/fmicb.2021.705501)
Supplement: Supplementary file 1 [file Data_Sheet_1.docx]

Supplementary Material

# TRACER EXPERIMENTS

A previous set of tracer experiments were done prior to the main experiments that were useful in determining the relative contributions of methanogenesis pathways. The experimental setup was the same as the main experiments, except PIPES was used as a buffer and the pH was not adjusted. However, instead of using sodium acetate with natural abundance ratios of ^13^C:^12^C, ^13^C-labeled sodium acetate with the C1 carbon labeled (^12^CH_3_^13^COO^-^). The RGA methods were similar to those used in the main experiments, except *m/z* 17 was used to measure ^13^CH­_4_ and *m/z* 45 was used to measure ^13^CO_2_. Calibration curves were not made for ^13­^CH_4_ and ^13­^CO_2_, so for the purposes of interpretation of these results, ^13^CH_4_ and ^13^CO_2_ were presumed to behave similar to their ^12^C counterparts. One replicate of control incubations was removed because it appeared to be an outlier, with little methane production compared to other control replicates in the tracer experiments as well as control experiments included in the main text.

The two major pathways of methane considered were acetoclastic methanogenesis (1) and hydrogenotrophic methanogenesis (2).

^12^CH_3_^13^COO^-^ + H_2_O→ ^12^CH_4(aq)_ + H^13^CO_3_^-^ (1)

¼H^13^CO_3_^-^ + H_2(aq)_ + ¼H^+^ → ¼^13^CH_4(aq)_ + ¾ H_2_O (2)

In the case of acetoclastic methanogenesis, essentially all CH_4_ would be ^12^CH_4_ because acetoclastic methanogenesis uses the methyl-carbon in acetate (Ferry, 1992)_._ In contrast, if hydrogenotrophic methanogenesis were occurring, the CH_4_ produced would have roughly the same isotopic signature as the CO_2_ that is being reduced to form the CH_4_, with differences only due to fractionation.

While exact proportions of ^13^C-labeled products could not be determined, the results can still be used to understand general trends. The ratio of *m/z* 17:15 stayed below 5:100, indicating that the methane produced was essentially exclusively ^12^CH_4_. Total ^12^CH_4_ produced was about 2 mmol (Supplementary Figure 5), which is about the same amount of CH_4_ production as non-tracer experiments in the main paper. Additionally, the ^13^CO_2_ content increased throughout the course of the experiment (Supplementary Figure 5), which is indicative of the carboxyl group of the acetate being converted into CO_2_. This supports methane production through acetoclastic methanogenesis. Because the conditions of these experiments were similar to the main experiments, the main experiments are also likely acetoclastic methanogenesis.

REFERENCES

Amend, J. P., and Shock, E. L. (2001). Energetics of overall metabolic reactions of thermophilic and hyperthermophilic Archaea and Bacteria. *FEMS Microbiol. Rev.* 25, 175–243. doi:10.1111/j.1574-6976.2001.tb00576.x.

Benner, S. G., Hansel, C. M., Wielinga, B. W., Barber, T. M., and Fendorf, S. (2002). Reductive dissolution and biomineralization of iron hydroxide under dynamic flow conditions. *Environ. Sci. Technol.* 36, 1705–1711. doi:10.1021/es0156441.

Bethke, C. M., Sanford, R. A., Kirk, M. F., Jin, Q., and Flynn, T. M. (2011). The thermodynamic ladder in geomicrobiology. *Am. J. Sci.* 311, 183–210. doi:10.2475/03.2011.01.

Bonneville, S., Van Cappellen, P., and Behrends, T. (2004). Microbial reduction of iron(III) oxyhydroxides: Effects of mineral solubility and availability. *Chem. Geol.* 212, 255–268. doi:10.1016/j.chemgeo.2004.08.015.

Cornell, R. M., and Schwertmann, U. (2003). *The Iron Oxides: Structure, Properties, Reactions, Occurrences and Uses*. 2nd ed. Wiley-VCH: Weinheim doi:10.1002/3527602097.

Cutting, R. S., Coker, V. S., Fellowes, J. W., Lloyd, J. R., and Vaughan, D. J. (2009). Mineralogical and morphological constraints on the reduction of Fe(III) minerals by Geobacter sulfurreducens. *Geochim. Cosmochim. Acta* 73, 4004–4022. doi:10.1016/j.gca.2009.04.009.

Ferry, J. G. (1992). Methane from acetate. *J. Bacteriol.* 174, 5489–5495. doi:10.1128/jb.174.17.5489-5495.1992.

Hansel, C. M., Benner, S. G., Neiss, J., Dohnalkova, A., Kukkadapu, R. K., and Fendorf, S. (2003). Secondary mineralization pathways induced by dissimilatory iron reduction of ferrihydrite under advective flow. *Geochim. Cosmochim. Acta* 67, 2977–2992. doi:10.1016/S0016-7037(03)00276-X.

Lovley, D. R., and Phillips, E. J. (1988). Novel mode of microbial energy metabolism: organic carbon oxidation coupled to dissimilatory reduction of iron or manganese. *Appl. Environ. Microbiol.* 54, 1472–80. Available at: http://www.ncbi.nlm.nih.gov/pubmed/16347658 [Accessed March 1, 2019].

Majzlan, J., Navrotsky, A., and Schwertmann, U. (2004). Thermodynamics of iron oxides: Part III. Enthalpies of formation and stability of ferrihydrite (∼Fe(OH)3), schwertmannite (∼FeO(OH)3/4(SO4)1/8), and ε-Fe2O3. *Geochim. Cosmochim. Acta* 68, 1049–1059. doi:10.1016/S0016-7037(03)00371-5.

Stumm, W. (Ed. ., and Morgan, J. J. (ed. . (1993). *Aquatic Chemistry: Chemical Equilibria and Rates in Natural Waters*. doi:10.1016/S0016-7037(97)81133-7.

# SUPPLEMENTARY FIGURES
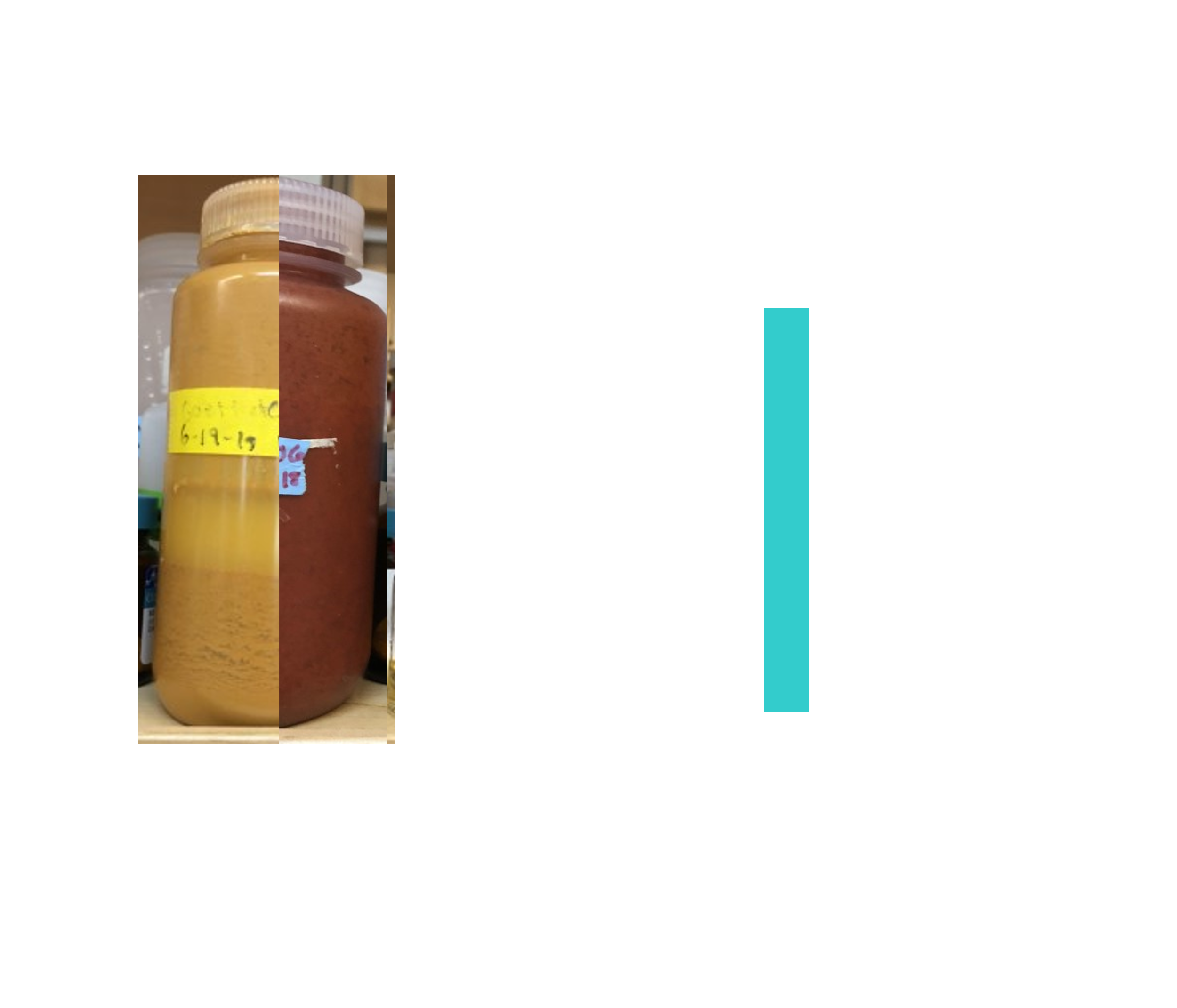


Supplementary Figure 1. Photos of the initial goethite (left) and hematite (right) to show color of mineral syntheses (A) and quantification of acid-extractable Fe from the first time point of incubation experiments (B)


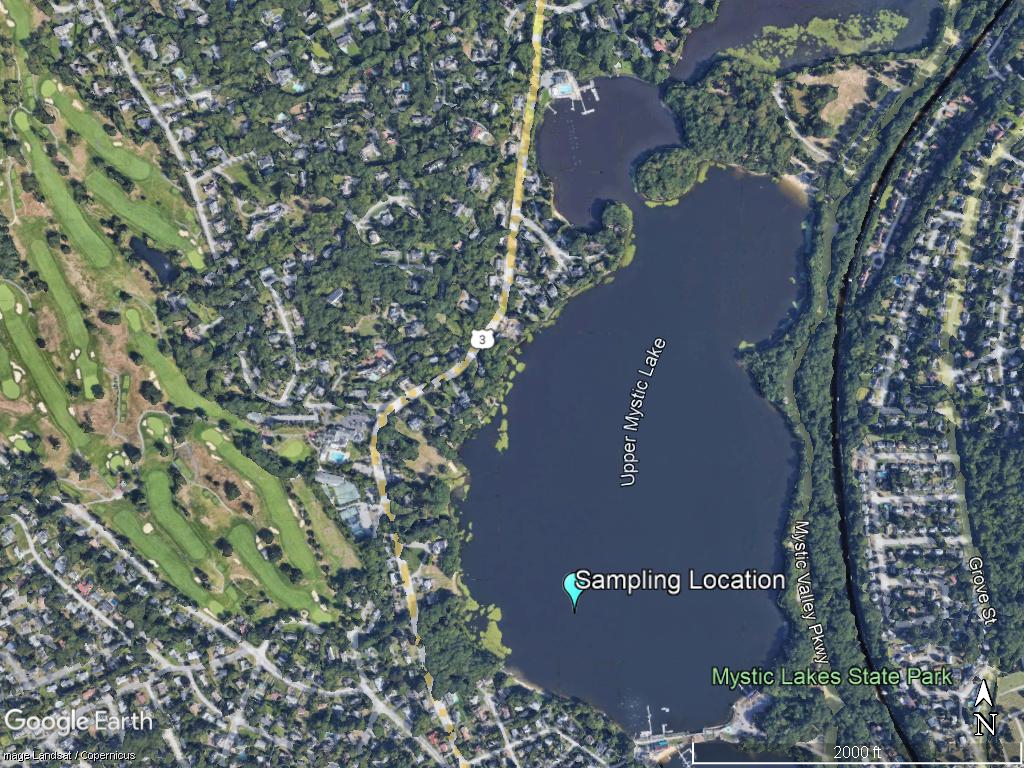


Supplementary Figure 2. Site of sediment water collection in Upper Mystic Lake

**
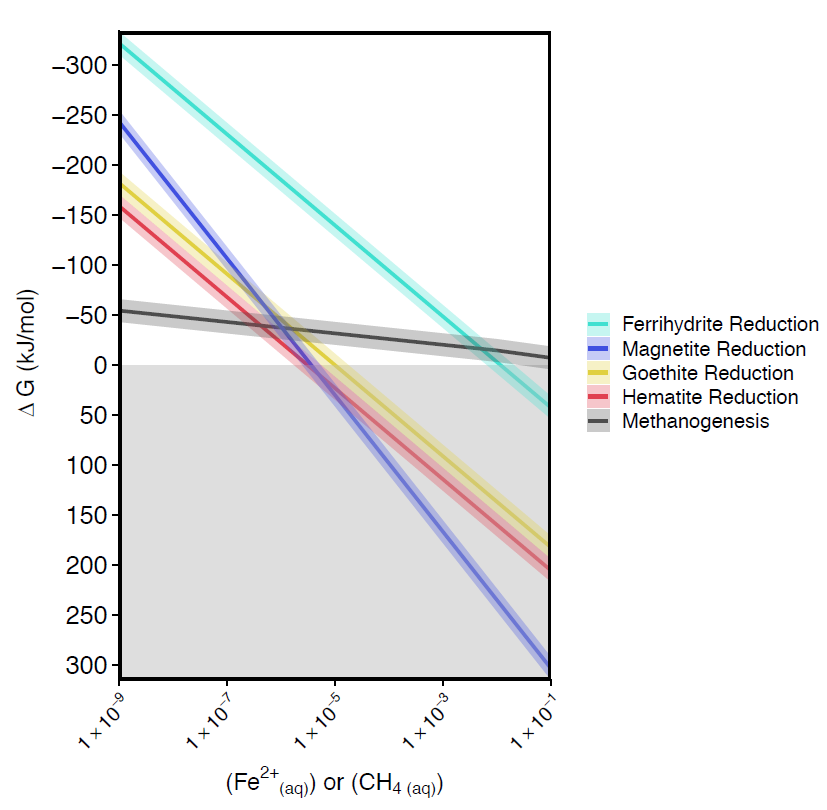
**

**Supplementary Figure 3. ∆G of acetate-consuming (reactions shown in Table 2 over a range of Fe^2+^_(aq)_ (for iron oxide reduction) and CH_4(aq)_ (for methanogenesis) activities. Calculations were done at pH 7 using (HCO_3_^-^) = 0.01. Gray shaded region represents ∆G > 0. Colored shaded regions surrounding reaction lines represent acetate activities from 10^-6^ (lower bound) to 10 (upper bound). Order of reactions listed in the legend from top to bottom correspond to lines from top to bottom where lines intersect the y-axis.**

Supplementary Figure 4. ^12^CH4 (A) and perfect ^12^CO2 as a percent of total CO_2_(B) produced over time. Error bars represent standard deviations of three replicates. Points without error bars have standard deviations smaller than the symbols

Supplementary Figure 5. Total observed ASVs (A), Simpson Index (B), and Shannon Index (C) in UML and experimental samples

Supplementary Figure 6. Abundance plots of 16s-rRNA data by phylum. Each bar is the average of three experimental replicates.

Supplementary Figure 7. Abundance plots of 16s-rRNA data by Genus (A) and ASV (B) for the phylum Bacteroidetes. Only the top 50 ASVs of Bacteroidetes are included in the legend. ASVs are listed in order from most to least abundant (sum total across all samples). Each bar is the average of three experimental replicates.

Supplementary Figure 8. Abundance plots of 16s-rRNA data by Genus (A) and ASV (B) for the phylum Firmicutes. Only the top 30 genera and the top 50 ASVs of Bacteroidetes are included in the legend. Genera and ASVs are listed in order from most to least abundant (sum total across all samples). Each bar is the average of three experimental replicates.

**

**Supplementary Figure 9. PCoA plot of microbial communities based on genus-level relative abundance data. Each time point for each experimental condition has 3 replicates represented separately**

**Supplementary Figure 10. Number of ASVs significantly different from control experiments at each time point separated within each bar by phylum classification.**

Supplementary Figure 11. Heatmap of top 100 ASVs with clustering based on PCoA ordination of samples. ASVs are labeled by family (left) and ASV# (right). ASVs left blank in A could not be classified at the family level. Red boxes highlight the cluster of ASVs that are highly abundant in ferrihydrite samples compared to all other samples.

# SUPPLEMENTARY TABLES

Supplementary Table 1. ∆G°_f_ values for reactants and products used for ∆Gº_rxn_ values in Table 2. Value for 2-line ferrihydrite is used for Fe(OH)_3_

| **Constituent** | **∆G°_f_ (kJ/mol)** | **Ref** |
| --- | --- | --- |
| Fe^2+^_(aq)_ | -78.87 | (Cornell and Schwertmann, 2003) |
| HCO_3_^-^ | -586.8 | (Stumm and Morgan, 1993) |
| CH_4(aq)_ | -34.39 | (Stumm and Morgan, 1993) |
| CH_3_COO^-^ | -369.33 | (Amend and Shock, 2001) |
| H_2(aq)_ | 17.57 | (Stumm and Morgan, 1993) |
| H^+^ | 0 | (Stumm and Morgan, 1993) |
| FeOOH | -488.8 | (Cornell and Schwertmann, 2003) |
| Fe_2_O_3_ | -746.2 | (Cornell and Schwertmann, 2003) |
| Fe(OH)_3_ | -708.5 | (Majzlan et al., 2004) |
| Fe_3_O_4_ | -1012.6 | (Cornell and Schwertmann, 2003) |
| H_2_O | -237.18 | (Stumm and Morgan, 1993) |

Supplementary Table 2. P-values of pairwise comparisons of group of samples based on mineral type using PERMANOVA. (** significant at p<0.01, * significant at p<0.05)

|  | **Control** | **Ferrihydrite** | **Goethite** |
| --- | --- | --- | --- |
| **Ferrihydrite** | 0.003** | - | - |
| **Goethite** | 0.019* | 0.003** | - |
| **Hematite** | 0.076 | 0.004 | 0.136 |

Supplementary Table 2. ∆G°_f_ values for reactants and products used for ∆Gº_rxn_ values in Table 2. Value for 2-line ferrihydrite is used for Fe(OH)_3_

| **Constituent** | **∆G°_f_ (kJ/mol)** | **Ref** |
| --- | --- | --- |
| Fe^2+^_(aq)_ | -78.87 | (Cornell and Schwertmann, 2003) |
| HCO_3_^-^ | -586.8 | (Stumm and Morgan, 1993) |
| CH_4(aq)_ | -34.39 | (Stumm and Morgan, 1993) |
| CH_3_COO^-^ | -369.33 | (Amend and Shock, 2001) |
| H_2(aq)_ | 17.57 | (Stumm and Morgan, 1993) |
| H^+^ | 0 | (Stumm and Morgan, 1993) |
| FeOOH | -488.8 | (Cornell and Schwertmann, 2003) |
| Fe_2_O_3_ | -746.2 | (Cornell and Schwertmann, 2003) |
| Fe(OH)_3_ | -708.5 | (Majzlan et al., 2004) |
| Fe_3_O_4_ | -1012.6 | (Cornell and Schwertmann, 2003) |
| H_2_O | -237.18 | (Stumm and Morgan, 1993) |

Supplementary Table 3. Fe speciation of HCl-extractable solid phase (as shown in Figure 2 in main text). A portion of the solid speciation could not be determined because the aqueous Fe speciation was not determined and these calculations were done based on the total HCl-extractable phase, which includes aqueous Fe

| **Time (days)** | **Fraction Fe(III) (Avg)** | **Fraction Fe(III) (Stdev)** | | **Fraction Fe(II) (Avg)** | **Fraction Fe(II) (Stdev)** | **Fraction Undetermined (Avg)** | **Fraction Undetermined (Stdev)** |
| --- | --- | --- | --- | --- | --- | --- | --- |
| **0** | 0.996811 | | 0.005524 | 0.003062 | 0.005303 | 0.000128 | 0.000221 |
| **3** | 0.774208 | | 0.093405 | 0.173571 | 0.078689 | 0.05222 | 0.018626 |
| **6** | 0.614418 | | 0.084808 | 0.212165 | 0.038511 | 0.173417 | 0.077052 |
| **9** | 0.407054 | | 0.022151 | 0.344142 | 0.046827 | 0.248804 | 0.024676 |
| **13** | 0.412141 | | 0.1429 | 0.374246 | 0.078034 | 0.213613 | 0.064866 |
| **16** | 0.487936 | | 0.125031 | 0.349208 | 0.097855 | 0.162856 | 0.036195 |
| **20** | 0.307948 | | 0.036235 | 0.515677 | 0.005664 | 0.176375 | 0.033971 |
| **24** | 0.262826 | | 0.051055 | 0.488096 | 0.057651 | 0.249078 | 0.097141 |
| **28** | 0.402045 | | 0.029683 | 0.490731 | 0.017196 | 0.107223 | 0.012486 |
| **32** | 0.322806 | | 0.031867 | 0.539258 | 0.044638 | 0.137936 | 0.061273 |
